# An overview of PROTACs: a promising drug discovery paradigm

**DOI:** 10.1186/s43556-022-00112-0

**Published:** 2022-12-20

**Authors:** Zi Liu, Mingxing Hu, Yu Yang, Chenghao Du, Haoxuan Zhou, Chengyali Liu, Yuanwei Chen, Lei Fan, Hongqun Ma, Youling Gong, Yongmei Xie

**Affiliations:** 1 State Key Laboratory of Biotherapy and Cancer Center, Department of Laboratory Medicine, West China Hospital, Sichuan University and Collaborative Innovation Center of Biotherapy, Chengdu, 610041 China; 2 Department of Biological Sciences, USC Dana and David Dornsife College of Letters, Arts and Sciences, Los Angeles, 90089 USA; 3 Hinova Pharmaceuticals Inc., Chengdu, 610041 China; 4 Department of Thoracic Oncology, West China Hospital, Sichuan University, Chengdu, 610041 China

**Keywords:** PROTAC, Protein degradation, Ubiquitin–proteasome system, Design and synthesis

## Abstract

Proteolysis targeting chimeras (PROTACs) technology has emerged as a novel therapeutic paradigm in recent years. PROTACs are heterobifunctional molecules that degrade target proteins by hijacking the ubiquitin–proteasome system. Currently, about 20–25% of all protein targets are being studied, and most works focus on their enzymatic functions. Unlike small molecules, PROTACs inhibit the whole biological function of the target protein by binding to the target protein and inducing subsequent proteasomal degradation. PROTACs compensate for limitations that transcription factors, nuclear proteins, and other scaffolding proteins are difficult to handle with traditional small-molecule inhibitors. Currently, PROTACs have successfully degraded diverse proteins, such as BTK, BRD4, AR, ER, STAT3, IRAK4, tau, etc. And ARV-110 and ARV-471 exhibited excellent efficacy in clinical II trials. However, what targets are appropriate for PROTAC technology to achieve better benefits than small-molecule inhibitors are not fully understood. And how to rationally design an efficient PROTACs and optimize it to be orally effective poses big challenges for researchers. In this review, we summarize the features of PROTAC technology, analyze the detail of general principles for designing efficient PROTACs, and discuss the typical application of PROTACs targeting different protein categories. In addition, we also introduce the progress of relevant clinical trial results of representative PROTACs and assess the challenges and limitations that PROTACs may face. Collectively, our studies provide references for further application of PROTACs.

## Introduction

**Fig. 1:**
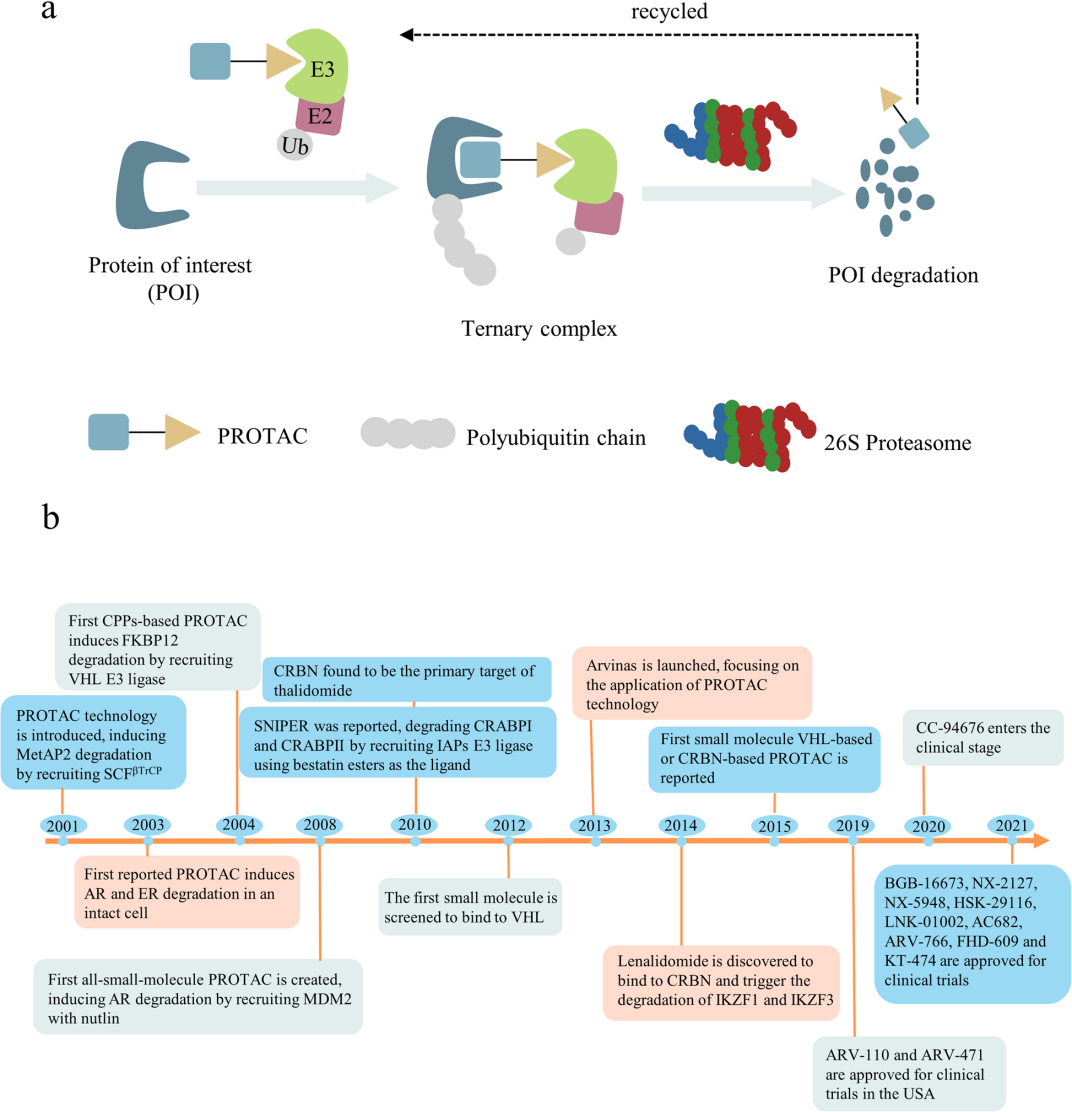
**a** The mechanism of PROTACs based on the UPS. UPS consists of specific enzymes (E1, E2 and E3) modifying proteins with ubiquitin and the proteasome degrading the ubiquitin-tagging proteins. PROTAC contains a POI ligand, an E3 ligand and a linker. The E3-PROTAC-POI ternary complex induces the polyubiquitination and proteasome-mediated degradation of POIs. **b** Milestones in the development of PROTAC technology

Proteolysis targeting chimeras (PROTACs) were first reported by Sakamoto et al. in 2001 [1]. PROTACs are heterobifunctional molecules that contain three components: the protein-of-interest (POI) binding moiety, a linker, and E3 ubiquitin ligase binding moiety (Fig. 1a) [2, 3]. PROTAC molecule can bind with E3 ligase and the target protein to form POI-PROTAC-E3 ligase ternary complex [4, 5]. Hijacking the ubiquitin-protease system (UPS) subsequently causes the target protein to be polyubiquitinated, which is then followed by the proteasomal degradation of protein. In eukaryotic cells, the UPS is the primary mechanism for maintaining protein homeostasis removing defective and damaged proteins [6, 7]. The UPS system degrades proteins by substrate-specific ubiquitination and recognition. Ubiquitination is a continuous three-step process that involves a cascade of three enzymes: ubiquitin-activating enzymes (E1), ubiquitin-conjugating enzymes (E2), and substrate-specific ligases (E3) [8–11]. E1 activates the free ubiquitin (Ub) in an ATP-dependent process by forming a ubiquitin-E1 thioester bond, and then E1 subsequently transfers the activated Ub to E2 via trans-thioesterification [12]. Finally, the Ub-tagged E2 and target protein are recruited by E3 ligase to facilitate ubiquitin labeling on target proteins [13]. Such ubiquitination processes can be recycled to generate poly-ubiquitin chain tagged target protein, which directs the marked protein to 26S proteasome to undergo degradation [14]. PROTACs simultaneously recruit E3 ligase and POI, bringing POI and E3 Ligase in spatial proximity. PROTACs simulate specific recognition of substrate by E3 ligase and hijack the intracellular protein destruction mechanism to remove POIs from cells [15].

With the assistance of modern molecular biology methods and human genome information, target-based approaches have been applied to drug discovery [16]. Modern drug discovery focuses on finding small molecules with high binding affinity to target proteins, which modulate protein function by occupying the enzymatic activity site [17]. However, some proteins lack bindable sites or enzymatic activity sites, like transcription factors, RAS family proteins [18], scaffolding proteins and regulatory proteins [19], making them insensitive to traditional small molecule drugs. With the advent of PROTACs, it is possible to degrade “undruggable protein” without taking the presence of the active sites into account. PROTACs broaden the horizon for future drug discovery with unique advantages. A series of PROTACs drug using for degradation of androgen receptors (AR) [20] and estrogen receptors (ER) [21] have already entered in phase II clinical trials. By far, the PROTACs have proved effective in degrading a variety of proteins, such as representative AR, ER nuclear receptors, various kinases, transcription factors, and abnormal protein aggregates. In 2001, Craig Crews and his coworker reported the first heterobifunctional molecule, and it consisted of angiogenesis inhibitor ovalicin recruiting methionine aminopeptidase-2 (MetAP-2) and IкBα phosphopeptide recruiting E3 ubiquitin ligase β-TRCP [1]. The F-box protein β-transducin repeat-containing protein (β-TRCP) has been demonstrated to bind to IкBα, a negative regulator of NF-кB. Studies had shown that this PROTAC can effectively reduce MetAP-2 levels in vitro. This groundbreaking work persisted after extracellular studies showed promise. In 2003, PROTACs were applied to targeted the degradation of the ER and AR receptors [22]. IкBα phosphopeptide was connected to estradiol and dihydrotestosterone (DHT) respectively, making PROTACs are potent for degrading Erα and AR. The first cell-permeable PROTAC was developed by Schneekloth et al*.* in 2004 and contained the E3 ligase binding peptide ALAPYIP to recruit von Hippel-Lindau (VHL) which could induce the degradation of AR and FK506 binding protein 12 (FKBP12) [23, 24]. Additionally, a poly-D-arginine tag was incorporated into the carboxy terminus of the peptide sequence to confer cell permeability and resist nonspecific proteolysis [23]. These works are pioneering examples of PROTACs with in vivo validity. Although peptide based PROTACs have the advantages of high biocompatibility and low toxicity in vivo, it is impossible to ignore the limited cell permeability and synthetic problem caused by the large molecular weight. Along with the development of small molecule ligand for E3 ligase such as mouse double minute 2 (MDM2) [2], cell inhibitor of apoptosis protein (cIAP) [25], VHL, Cereblon (CRBN) [26], DCAF11 [27], DCAF15 [28], DCAF16 [29], KEAP1 [30], and RNF114 [31], PROTACs go into small molecularization rapidly [32]. The SARM-nutlin PROTAC was the first all-small molecule PROTAC that consisted of three parts: a specific substrate of AR, a ligand binding to E3 ligase MDM2, and a short soluble polyethylene glycol (PEG) linker joining these two moieties [2]. The in vitro results revealed that the SARM-nutlin PROTAC was capable of inducing AR proteasomal degradation and this is a significant improvement to the PROTACs.

**Fig. 2:**
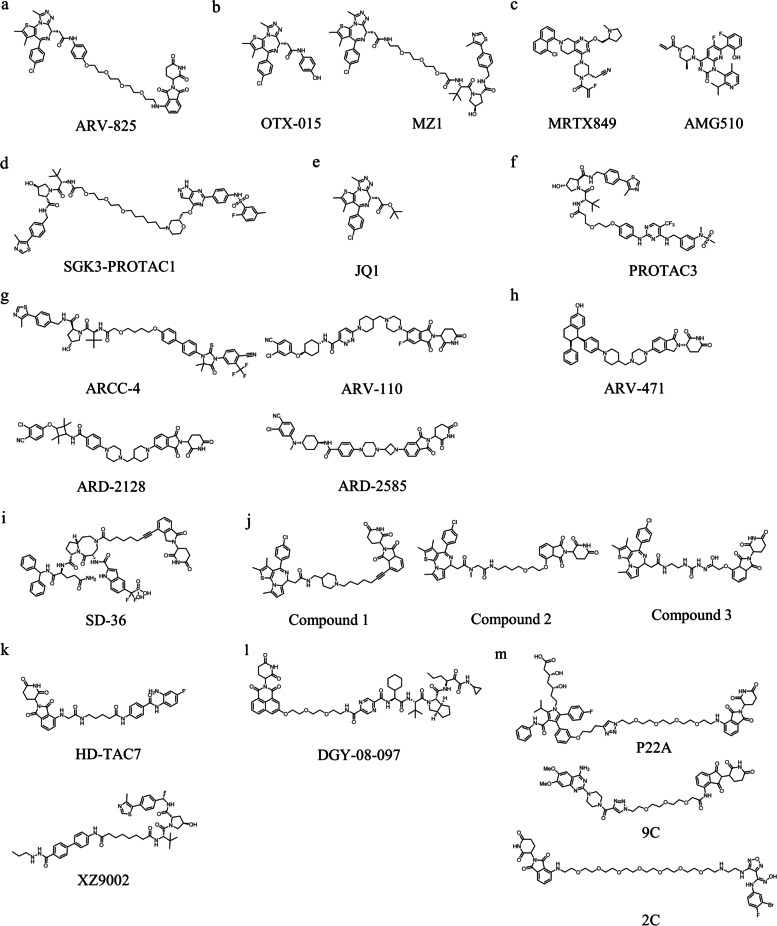
The representative PROTACs targeting diverse proteins

Research on the CRBN E3 complex led to vital breakthroughs in 2010. CRBN, a substrate receptor subunit of CUL4-RBX1-DDB1-CRBN (CRL4^CRBN^) E3 ubiquitin ligase [33], has been identified as the direct target for thalidomide immunomodulatory drugs (IMiDs) [34]. These IMiDs bind CRBN ligase, which provided a binding site for multiple transcription factors with zinc finger domain such as IKZF1 and IKZF3, forming the basis of their anti-cancer effects [35–38]. Over the past few years, thalidomide and its derivatives have been successfully applied in PROTACs for target degradation of various protein classes for its efficient POI degradation and promising druggability [39]. ARV-825 (Fig. 2a) was the first PROTAC that incorporated pomalidomide and Bromodomain-containing protein 4 (BRD4) inhibitor OTX015 (Fig. 2b) [26]. This PROTAC could degrade BRD4 protein in Burkitt’s lymphoma (BL) cells effectively.

In 2012, Crews and Ciulli team identified the first small molecule ligand for VHL with micromolar dissociation constant [40]. To improve the affinity and lipophilicity of the first generation VHL ligand, Galdeano et al*.* discovered the second-generation ligands for VHL with the key hydroxyproline (Hyp) group as core recognition motif, VH032 was identified as the most potent ligand for VHL with nanomolar affinity. Identification of the novel VHL E3 ligands marked a milestone in PORTACs technology (Fig. 1b). These newly discovered VHL ligands opened up new opportunities to design VHL-recruiting PROTACs. The crystal structure of VH032 coupled with VHL was solved by Zengerle et al., providing the structural details for constructing the first VHL-recruiting PROTACs, MZ1 (Fig. 2b). Compound MZ1 potently and selectively removed BRD4 over BRD2 and BRD3. Gadd et al*.* later uncovered the crystal structure of the BRD4-MZ1-VHL complex, revealing MZ1 was “sandwiched” between BRD4 and E3 ligase and the new contact of BRD4-VHL is generated by MZ1-induced cooperative recognition, they found that PROTAC-induced electrostatic surface interactions between the target protein and E3 ligase are important for stabilizing the ternary complex [5]. The aforementioned groundbreaking research lay the foundation for later VHL-based PROTACs studies. To date, VHL-based PROTACs have successfully applied to the degradation of various disease-associated proteins, such as BCR-ABL [41], ALK (anaplastic lymphoma kinase) [42], and FAK (Focal adhesion kinase) [43].

PROTAC technology has been applied to the alternative treatment of various diseases. More and more targets have been confirmed to be “PROTACable” genome, and some PROTAC molecules have achieved clinical benefits. For instance, PROTAC targets that have entered the clinical trials include AR [44], ER [45], IRAK4, STAT3, BTK, BRD9, BCR-Xl [46], etc. There are at least 20 PROTACs in the clinical trials by the end of 2022 (Table 1), with more expected to follow. Among them, ARV-110 and ARV-471 from Arvinas are the most advanced PROTAC drugs in clinical research and have entered the clinical phase II study. Arvinas, C4 therapeutics, Kymera Therapeutics, and Captor Therapeutics are pioneering pharmaceutical companies in the field of PROTACs, which promote the clinical translation of PROTACs. Arvinas is dedicated to advancing its representative ARV-110 and ARV-471 into the market. Early clinical data of ARV-110 and ARV-471 demonstrated ideal safety, effective exposure and meaningful clinical efficacy for patients, proving the therapeutic feasibility of the approach. The research data indicate that ARV-110 is safe as an oral bioavailable degradation agent. Phase I trials have shown that ARV-110 reduced prostate-specific antigen (PSA) levels by more than 50% in 40% of patients with mCRPC in a population with a specific gene mutation. In addition, in the initial clinical study, the biopsy data of one patient showed a 70% ~ 90% decrease in AR. A Phase I clinical study of ER+ and HER2- breast cancer patients who had received an average of five continuous treatments showed that ARV-471 could significantly reduce the expression level of ER in tumor tissue of patients, reducing the ER level by 62% on average, up to 90% at most. In addition, the phase I clinical data of ARV-471 also showed that a high level of ER degradation (89%) was observed at all dose levels of 30-700 mg, and it was well tolerated. ARV-471 exhibited certain degradation effects on both wild type ER and ER mutants. ARV-471 is undergoing a phase II dose expansion clinical trial to evaluate the efficacy of ARV-471 in the treatment of ER + /HER2- patients with locally advanced or metastatic breast cancer.

**Table 1:** The summary of selected degraders in and approaching the clinical

Drug	Target	Sponsor	Disease	Phase
ARV-110	AR	Arvinas	Metastatic Castration Resistant Prostate Cancer	Phase 2
ARV-766	AR	Arvinas	Metastatic Castration Resistant Prostate Cancer	Phase 1
CC-94676	AR	Celgene	Metastatic Castration Resistant Prostate Cancer	Phase 1
GT-20029	AR	Kintor	Prostate Cancer	Phase 1
HP518	AR	Hinova	Metastatic Castration-Resistant Prostate Cancer	Phase 1
ARV-471	ER	Arvinas	ER + /HER2- Locally Advanced or Metastatic Breast Cancer	Phase 2
AC682	ER	Accutar Biotech	Locally Advanced or Metastatic ER + Breast Cancer	Phase 1
DT-2216	BCR-xL	Dialectic	Solid tumor/Hematologic malignancy	Phase 1
KT-474	IRAK4	Kymera	Atopic Dermatitis (AD) or Hidradenitis Suppurativa (HS)	Phase 1
KT-413	IRAK4	Kymera	Diffuse Large B Cell Lymphoma	Phase 1
KT-333	STAT3	Kymera	Solid Tumor, Hematologic Malignancies	Phase 1
NX-2127	BTK	Nurix	B-cell malignancies	Phase 1
NX-5948	BTK	Nurix	B-cell malignancies/ Autoimmune diseases	Phase 1
BGB-16673	BTK	BeiGene	B-Cell malignancies	Phase 1
HSK-29116	BTK	Haisco	Relapsed/Refractory B-cell malignancies	Phase 1
CFT8634	BRD9	C4 Therapeutics	Synovial Sarcoma	Phase 1/2
FHD-609	BRD9	Foghorn Therapeutics	Advanced Synovial Sarcoma	Phase 1
CFT8919	EGFR L858R	C4 Therapeutics	Non-small-cell Lung Cancer	IND
LNK-01002	Ras GTPase	Lynk	Primary (PMF) or Secondary Myelofibrosis (PV-MF, ET-MF) or Acute Myeloid Leukemia	IND
CG001419	TRK	Cullgen	Cancer and other indications	IND

Data source: https://clinicaltrials.gov updated: 9/30/2022

*IND* Investigational New Drug

The clinical trials results of the most advanced PROTAC drugs ARV-110 and ARV-471 were considered as weathervane for the development of PROTACs field. As a new drug paradigm, there are still considerable questions about the clinical transformation of PROTAC drugs. How PROTACs ensure therapeutic effects without meeting the Lipinski’s Rule-of-Five, and how to study, elucidate and minimize the complex off-target effects and side effects that might be caused by the heterobifunctional molecule form of PROTAC drug are unavoidable challenges in clinical trials. As the first two PROTAC drugs to enter phase II clinical trials, ARV-471 and ARV-110 have shown strong clinical performance in the early trials. If the results of the subsequent phase II and phase III clinical trials can achieve the expected goals, PROTAC may enter clinical use in the near future, which will bring a historic breakthrough in the research and development of targeted protein degradation drugs.

In this review, we list the targets that PROTACs have been applied for the first time in the last 5 years (Table 2). We briefly recount the advantages of PROTAC technology compared to other technologies. We then outline the recent progress of PROTACs for targeting diverse related proteins, especially those in clinical trials. The essential considerations for designing a new PROTAC molecule were also discussed and recommended. We try to provide a valuable reference for people in the related fields to design potent PROTACs.

**Table 2:** PROTACs first reported in the last 5 years (2017–2022)

Targets	Year	Reference
AKT	2020	[47]
Alpha-syn, SNCA, NACP	2020	[48]
Alpha-tubulin, TUBA	2020	[49]
AXL, UFO	2020	[50]
BCL2	2019	[51]
BCL-xL	2019	[52]
Beta-tubulin, TUBB	2020	[49]
BLK	2020	[53]
BRD2 BD2	2018	[54]
BTK	2018	[55]
Cdc20, p55CDC	2019	[56]
CDK2, CDKN2	2020	[57]
CDK4, PSK-J3	2019	[58]
CRBN	2018	[59]
CYP1B1	2020	[60]
EED	2020	[61]
EGFR, ERBB, HER1	2018	[62]
EZH2, KMT6, ENX-1	2020	[61]
FAK, PTK2, FAK1	2018	[63]
HDAC3	2020	[64]
IDO1	2020	[65]
MDM2	2019	[66]
Tau	2019	[67]
VHL, pVHL	2017	[68]
Wee1, WEE1hu	2020	[69]
HER2	2022	[70]

## The advantages of PROTACs

Diverse novel therapeutic strategies (e.g., small-molecule inhibitors, monoclonal antibodies and RNA interference (RNAi)), have become a well-established paradigm for drug discovery. The activity of small molecule-inhibitors usually depend on occupying the active pocket of the target, competing with endogenous ligands to inhibit the function of the target protein or enzyme. Long-term clinical application of small-molecule inhibitors faces the challenges of drug resistance and off-target effects [71]. Monoclonal antibody drugs regulate cellular responses by blocking extracellular protein–protein or protein–ligand interactions. The major advantages of monoclonal antibody drugs stem from their high affinity to the target protein, whereas, the deficiencies of the monoclonal antibody drugs involve their poor cell permeability, oral unavailability, and high cost. RNA interference is used to induce gene silencing by knocking down mRNA, due to the catalytic nature of RNAi, which is capable of degrading multiple equivalents of mRNA transcripts. However, the off-target effects, poor oral bioavailability, and unsatisfactory tissue penetration made drug delivery challenging to study [72]. As a promising therapeutic paradigm, PROTACs have unique advantages over small-molecule inhibitors, monoclonal antibodies and other therapeutic strategies (Table 3) [73]. When the target protein is degraded by the proteasome, PROTACs can disassociate from the complex and continue to exert the degradation effect (called “event-driven” mechanism), allowing low exposures to be efficacious. Additionally, PROTACs completely abolish the target’s functionalities, and even ligands with lower POI/E3 affinity can be employed for target degradation. In this section, we will briefly compare PROTACs with other therapeutic strategies.

**Table 3:** Comparisons of PROTACs with other therapeutic strategies

	Small-molecule-inhibitor	Monoclonal antibody	PROTAC	RNA interfering
Intracelluar targets	+ +	-	+ +	+ +
Tissue penetration	+ +	+	+ +	+
Requirement of active sites	+ +	+ +	-	-
Undruggable targets	-	+ +	+ +	+ +
Catalytic mechanism of action	-	-	+ +	+ +
Elimination pathogenic proteins	-	-	+ +	+ +
Oral bioavailability	+ +	-	+ +	-
Selectivity	+	+ +	+ +	+ +
Catalytic MOA	-	-	+ +	+ +

“ +  + ” represents “Yes”, “ + ” represents “Poor”, “-” represents “No”

### Degrading “undruggable” proteins

Although FDA has approved nearly 400 drugs targeting human proteins, there are about 3000 disease-related proteins which are far more than we can handle [74]. However, most of them do not have appropriate therapeutic drugs, because the lack of so-called druggable deep grooves and active pockets to occupy for small molecules [75], such as scaffolding proteins, transcriptional factors, and RAt Sarcoma (RAS) proteins, are deemed “undruggable” proteins for a long time [76]. Therefore, it is difficult to regulate these undruggable proteins by small molecules that can only rely on continuous occupancy of the binding pocket of the target protein to exert their pharmacological activity (called “occupancy-driven” mechanism) [77–79]. Fortunately, PROTAC-induced protein degradation has the potential to address these issues. Unlike traditional small-molecule inhibitors, PROTACs do not require high affinity for ligands and long lasting occupancy, and even low affinity ligands can induce efficient degradation of target proteins [80]. For these challenging undruggable targets, PROTACs can bind to targeted proteins without the existence of active pockets, thus leading to proteasome mediated degradation and complete inhibition of the biological functions of target proteins [81]. For example, RAS proteins are the most frequently mutated oncoproteins in the lung, colorectal, and pancreatic cancers [82]. RAS proteins comprise three isoforms, KRAS, NRAS and HRAS [83]. Among them, KRAS mutation is a deadly driver of cancers. Due to the lack of a well-defined binding pocket, KRAS has been viewed as an undruggable protein for many years. However, FDA fast-tracked the designation of two covalent inhibitors, AMGEN’s sotorasib (AMG 510) and Mirati Therapeutics’ adagrasib (MRTX849) (Fig. 2c), both of which demonstrated potent inhibition of KRASG12C in clinical trials. Unfortunately, long term and prolonged usage inevitably results a severe decrease in affinity and acquired drug resistance [84]. In 2020, Crews et al. designed and synthesized a series of KRAS^G12C^ PROTAC via tethering MRTX849 with VHL ligands. After a degradation activity screen, they identified one of the most potent PROTAC that rapidly induced the degradation of KRAS^G12C^ protein (DC_50_ = 0.59 μM, NCI-H2030 cells) and also exhibited degradation activity in other cells [85].

### Improving selectivity and specificity

The main objective of medicinal chemistry researchers is the discovery of molecules with high selectivity to minimize adverse effects and toxicity brought on by off-target effects. However, it is difficult to achieve because of limited differences between proteins in the same family. Subtle differences in amino acid residues between the same family proteins are insufficient to provide adequate resolution for small molecular inhibitors. The unique mechanism of PROTAC endows it with the characteristics of dual selective substrate recognition. That is, in addition to the substrate selectivity of target protein ligands, the formation of stable POI-PROTAC-E3 ternary complexes before degradation also requires appropriate protein–protein interaction (PPI) between E3 ligase and target protein. Thus, selective recognition of target proteins from the whole protein level by E3 ligase improves the selectivity and specificity of PROTACs. A typical example of kinase isoform selectivity is targeting serum and glucocorticoid-induced protein kinase (SGK) and the SGK family contains three isoforms, SGK-1, SGK-2 and SGK-3. It was reported that mutant phosphoinositide 3-kinase (PI3K) can induce tumorigenesis through SGK3-dependent mechanism [86]. Some pieces of evidence suggest that various ATP-competitive inhibitors lack selectivity for all SGK isoforms, as they share similar affinity for different isoforms [87, 88]. However, the similar catalytic domain of the same family members prevented researchers from developing isoform-specific inhibitors. [89]. To address this problem, the highly specific SGK3-PROTAC1 (Fig. 2d) was developed. This PROTAC was designed by Tovell’s group based on the non-SGK3 selective inhibitor 308-R, to degrade SGK3 specifically [90]. At a low micromolar concentration of SGK3-PROTAC1, intracellular SGK3 levels can be significantly reduced without affecting SGK1 and SGK2. It could be assumed that the selectivity and specificity of SGK3-PROTAC1 derives from the selective recognition of SGK3 by VHL during the formation of ternary complexes induced by SGK3-PROTAC1.

### Catalytic mode of action (MOA)

Traditional small-molecule inhibitors act in a dose-dependent manner, to achieve clinical effect by maximizing drug-receptor occupancy. Excessive drug concentrations lead to undesirable side effects and off-target effects [91]. PROTACs can initiate the degradation of target protein catalytic and escape from proteasome [92]. Theoretically, PROTACs can be delivered at lower doses, for longer dosing intervals, and with lower toxicity than small molecule inhibitors since their low concentration is sufficient to degrade proteins and is not constrained by equilibrium occupancy. Because of their catalytic nature, low doses of PROTACs may reduce the probability of off-target effects to occur [77].

### Eliminate the accumulation of drug targets

The binding of small-molecule inhibitors to target proteins cansues increased protein accumulation even in a relatively short amount of time [93]. It can be attributed to two reasons: 1). drug binding to target proteins can stabilize the protein structure, thereby extending their half-life, and 2). long-term inhibition will cause upregulation of its compensatory expressio. In general, the accumulation of target protein can be detrimental to the efficacy of drugs. Therefore, for these proteins that are insensitive to inhibitors, it’s extremely suitable to take PROTAC-mediated protein degradation. For example, BRD4, as one of the important bromodomain and extraterminal domain (BET) family members [94]. Researchers demonstrated that targeting BRD4 is an effective means of suppressing MYC-driven cancers [95]. However, the small molecule BRD4 inhibitor, JQ1 (Fig. 2e) and OTX015 resulted in robust protein accumulation, and high concentration of inhibitor is required to suppress downstream c-MYC. In 2015, Lu et al. designed a potent BRD4 PROTAC (ARV-825) by hijacking CRBN E3 ligase, which induced a rapid and sustained degradation of BRD4 protein in all BL cell lines [26]. This highlights the advantages of PROTAC over small-molecule inhibitors.

### Others

In addition to the points mentioned above, PROTACs also have other advantages. The occurrence of acquired drug resistance is often closely related to point mutations that can decrease the affinity of the inhibitor to the target protein. PROTACs are able to overcome drug resistance issues via the complete elimination of the target mechanism [96]. Besides, the event-driven model of PROTACs do not require high drug exposure to reduce the risk of off-target effects [97]. Unlike other DNA-level protein knockout techniques, PROTACs enable for the rapid degradation of target proteins in vivo at the post-translational level. In the field of targeted protein degradation (TPD), besides UPS based PROTACs, lysosome-targeting chimeras (LYTACs), autophagy-targeting chimeras (AUTACs), and antibody-based PROTACs (AbTACs) degrade target proteins through lysomal. PROTACs cannot degrade extracellular and membrane proteins. Therefore, lysosome induced protein degradation can compensate for the lack of PROTACs. LYTACs were first proposed by Banik et al. and consist of a ligand binds lysosome-targeting receptors (LTRs) and a ligand binds extracellular or membrane protein [98]. Currently, only poly-serine-O-mannose-6-phosphonate (M6Pn) and N-acetyl galactosamine (Tri-GalNAC) were LTRs ligands available [99]. The LYTACs have been used to successfully degrade apolipoprotein E4, epidermal growth factor receptor (EGFR), programmed death protein ligand 1 (PD-L1), and CD71 [99]. However, due to the large molecular weight, poor cell permeability, and the possible emergence of immune response in vivo, further studies are needed [100]. In 2019, Takahashi et al. developed AUTACs based on the autophagic process for the degradation of endogenous proteins [101]. AUTACs are a bifunctional molecule with a linker joints POI ligand and autophagic recruitment tag. However, currently published AUTACs are inefficient due to the lack of efficient autophagy pathway recruiters. The autophagic process is extremely complex and may have an impact on natural autophagy, the mechanism of action of AUTACs remain unclear, so it need to be studied in depth [100]. AbTACs utilize bispecific antibodies, with one arm targeting POIs and the other targeting RNF43 E3 ligases [102]. AbTACs can induce POIs internalization and subsequent lysosomal degradation, but the the exact degradation mechanism remains to be confirmed.

## The typical application of PROTACs for targeting diverse proteins

In theory, PROTACs can degrade almost all intracellular proteins if there is an appropriate small molecule that specifically binds with those POI, but not all degraders outperform small-molecule inhibitors. Here, we summarize some typical PROTAC molecules that have demonstrated obvious inhibition activities, several of which have advanced to the clinical trial stage.

### PROTACs for targeting protein kinases

The human genome encodes over 500 protein kinases [103], making it the largest protein family. Currently, traditional small-molecule inhibitors are the primary treatment options for protein kinases related diseases. A majority of kinase inhibitors focused on the inhibition of receptor tyrosine kinase (RTK) [104]. However, the emergence of drug resistance impaired the clinical benefit, so it is urgent to apply novel therapeutic strategy to overcome this challenge.

In 2013, Crews’s group reported the earliest kinase PROTACs, which was used to target PI3K to block the human epidermal growth factor receptor 3 (ErbB3)–PI3K-Akt (protein kinase B) signal pathway [105]. This PROTAC is composed of two heterospecific peptide sequences recruiting POI and E3 ligase. An ErbB3-derived sequence that can bind to PI3K after it has been phosphorylated. Another sequence derived from hypoxia-inducible factor-1α (HIF1α) can be identified by VHL [105]. The two moieties were conjugated by a PEG linker, and a cell-penetrating sequence was incorporated to improve cell permeability. However, this PROTAC only display moderate potency because of poor permeability and unstable linker [106].

FAK, a tyrosine kinase, regulates many aspects of tumor progression (e.g., invasion, metastasis, and angiogenesis). The leading FAK kinase inhibitor defactinib, failed in clinical trials to treat malignant pleural mesothelioma stem cancer for the lack of efficacy. FAK also has a scaffolding role other than kinase, but kinase inhibitors cannot inhibit kinase-independent function. Cromm et al. designed PROTAC-3 (Fig. 2f) which could effectively induce the degradation of FAK with the IC_50_ of 6.5 nM [43]. PROTAC-3 is a bifunctional molecule consisting of defactinib and VHL ligand. It effectively inhibits FAK kinase-independent signaling and kinase-dependent signaling by efficient induction of degradation.

Bruton’s tyrosine kinase (BTK) is a member of the non-receptor cytoplasmic tyrosine kinase of the TEC family and a key regulator of the B cell receptor (BCR) signaling pathway, which plays a critical role in the life activities of B-cells like proliferation, survival, and differentiation [107, 108]. BTK is widely expressed in B cell neoplasms, and the clinical interventions are generally performed by inhibiting the kinase activity of BTK [109]. In 2013, FDA approved the first-in-class covalent inhibitor ibrutinib for the treatment of several B-cell malignancies. Ibrutinib binds covalently to Cysteine481 (C481) of BTK with IC_50_ of 0.5 nM [110, 111]. However, it has been revealed that a cysteine to serine mutation at position 481 of BTK (C481S) is what causes acquired resistance to ibrutinib [112]. So, induction of BTK protein degradation using PROTAC technology has emerged as a promising alternative approach. To date, four BTK degraders have entered clinical trials. They are NX-2127 (NCT04830137) and, NX-5948 (NCT05131022) from Nurix Therapeutics, HSK-29116 (NCT04861779) and BGB-16673 (NCT05006716) small molecule drugs from Haisco and BeiGene respectively. NX-2127 is an oral dual-target small molecule that possesses the activity of BTK degrader and IMiD neosubstrates degrader. A phase I clinical trial of NX-2127 is currently underway for the treatment of relapsed or refractory B-cell malignancies. Preclinical data have demonstrated that NX-2127 could potently induce the degradation of both ibrutinib-sensitive BTK^WT^ (wild type) and ibrutinib-resistant BTK^C481S^ in multiple cancer cell lines and human peripheral blood mononuclear cells (PBMCs) with the DC_50_ < 5 nM. Additionally, NX-2127 inhibited cell proliferation of BTK^C481S^ in TMD8 cells more effectively than ibrutinib. NX-2127 exhibits immunomodulatory activity through comprised of thalidomide IMiD [113]. Krönke et al*.* revealed that lenalidomide causes selective ubiquitination and degradation of CRBN neosubstrates Aiolos (IKZF3) and Ikaros (IKZF1) [35]. Lazarian et al*.* have shown that the overexpression of IKZF3 is a driver of BTK inhibitor resistance in chronic lymphocytic leukemia (CLL) [114]. Therefore, NX-2127 combines BTK degradation with IKZF degradation is expected to enhance its anti-tumor activity. NX-5948 is another BTK degrader designed by Nurix Therapeutics. Unlike NX-2127, NX-5948 lacks immunomodulatory activity and has the ability to cross the blood brain barrier (BBB) in animal models. NX-5948 displayed similar performance that preclinical data have shown that NX-5948 induced the degradation of BTK (50% degradation efficiency at < 1 n M) in lymphoma cell lines and PBMCs [115].

### PROTACs for targeting nuclear receptors

Nuclear receptors (NRs) belong to the family of transcription factors. Unlike other traditional transcription factors, its main function is to convert external the signal to transcriptional output [21]. A typical NR includes three domains: two structural domains that bind DNA and ligand respectively, and an unstructured N-terminal regulatory domain that is highly variable in terms of both sequence and size [116]. Ligand agonist binding confers a conformational change that results in exposure of the nuclear localization signal (NLS), which allows NR to translocate to the nucleus and bind the response elements. Small-molecule inhibitors that bind to ligand binding domain have been designed to activate or block the signal transduction function of nuclear receptors. However, small-molecule inhibitors have several disadvantages. For instance, our understanding of the concept of pure inhibitors is not clear, as continual AR antagonists prove to be agonists when the AR gene is overexpressed or mutated [117, 118]. In addition, some ligands for orphan NRs have not yet been identified, thus making it more complicated to target NRs to treat diseases. The advent of PROTAC technology has made it possible to target a wider range of NRs. NRs such as AR and ER participate in various important physiological progress in the body, and are closely related to prostate cancer and breast cancer. Therefore, a series of PROTACs targeting ER or AR have been developed.

AR signaling is critical in the development and maintenance of the normal function of prostate. AR not only plays a key role in the maintenance of musculoskeletal and male sex-related functions but also in the progression of prostate cancer [119]. Inhibition of AR function with AR antagonists such as enzalutamide and apalutamide is a common strategy in the treatment of prostate cancer [120]. Unfortunately, castration-resistant eventually occurs in patients with antiandrogen therapy [121]. PROTACs emerged as an alternative potential therapeutic approach to compensate for the shortcomings of AR inhibitors. Salami et al*.* synthesized a potent AR PROTAC ARCC-4 (Fig. 2g), which comprised of enzalutamide derivative and E3 ligand recruiting VHL. Compared with its parent inhibitor enzalutamide, ARCC-4 can effectively degrade AR and AR mutants caused by long-term use of clinical inhibitors, without leading to the presence of drug resistance [118]. It is well-known that ARV-110 (Fig. 2g) is the first AR-targeting PROTAC in clinical trial. The latest clinical trial data indicated that ARV-110 has an acceptable safety profile. The maximum tolerated dose (MTD) has not been established and the determination of the recommended phase 2 dose (RP2D) continues. In addition, ARV-110 has demonstrated antitumor activity in patients with metastatic castrate-resistant prostate cancer (mCRPC) following enzalutamid and/or abiraterone administration [44]. Recently, Wang’s group reporteded two highly potent and orally bioavailable AR PROTACs, ARD-2128 and ARD-2585 (Fig. 2g). ARD-2128 features an optimized AR antagonist linked to thalidomide via a rigid linker, achieving 67% oral bioavailability and better antitumor activity than enzalutamide in mice [122]. ARD-2585 incorporates the same CRBN ligand as ARD-2128 and achieves DC_50_ values of ≤ 0.1 nM in the VCaP cell line and 51% of oral bioavailability in mice [123].

Breast cancer is a malignant tumor in which the breast tissue becomes cancerous and the patient is usually a female population. Breast cancer can be subdivided into three types based on the status of the tumor receptor: estrogen receptor-positive (ER +), human epidermal growth factor receptor 2 positive (HER2+), and triple-negative subtypes (ER-, PR-,HER2-) [124]. Among these, ER + breast cancer is most commonly diagnosed [125]. ER is a member of nuclear receptor family, and ERα and ERβ regulate the gene expression of estrogen. Nevertheless, ERα has been verified to be primarily responsible for converting the estrogen signaling in the female reproductive system and mammary tissue [126, 127]. Most selective estrogen receptor degraders (SERD) were designed to target ERα to treat ER + breast cancer [128]. SERD is a class of small molecules that bind with ERα and subsequently degraded by proteasome. Fulvestrant is the only SERD that has been approved and administered by monthly intramuscular injection for the treatment of postmenopausal women with breast cancer [129–132]. To address the shortcomings of poor oral bioavailability of fulvestrant [131], a series of SERD molecules have been developed. However, SERD molecules could not degrade ER completely, and long-term use can lead to drug resistance. PROTAC technology offers an alternative treatment option [133–136]. Arvinas developed an ER-targeting PROTAC, ARV-471 (Fig. 2h), which was approved by the FDA to enter clinical trial for the treatment of patients with locally advanced or metastatic ER-positive/HER2-negative breast cancer [137]. Results from the mid-stage trials revealed that ARV-471 markedly reduced the expression level of ER in tumor tissues by an average of 62% and up to 90%. In addition, ARV-471 may degrade wild-type and clinically relevant ERα mutants (Y537S and D538G) with DC_50_ values of about 2 nM in multiple ER-positive breast cancer cell lines [138].

### PROTACs for targeting transcriptional factors

Transcriptional factors (TFs) are a class of proteins binding to DNA specific sequence to regulate gene transcription process [139]. TFs play a key role in multiple cell functions such as proliferation, differentiation and death. With the exception of nuclear receptors, direct targeting of transcription factors is particularly challenging for small-molecule inhibitors, thus rendering them considered as “undruggable protein” for decades [140–142]. Therefore, inducing protein degradation emerges as a potential modality for TFs [143]. Based on the specific structure of DNA-binding domains, TFs could be classified into tens of families [139]. It is worth noting that the C_2_H_2_ zinc­finger, homeodomain and helix-loop-helix families account for over 80% of the total number of transcription factors [140].

Signal transducer and activator of transcription 3 (STAT3) is a key nuclear transcription factor that is phosphorylated on tyrosine 705 and integrates cytokine and growth factor signaling to regulate an array of cellular process [144, 145]. STAT family comprises seven proteins, among which STAT3 has been shown to be overexpressed in many types of cancer, especially breast cancer. Targeting STAT3 is a prevalent strategy for the treatment of various cancers, inflammatory and autoimmune disorders [146]. The phosphorylation of STAT3 at Tyr705 can trigger its dimerization and is closely related to the transcriptional regulation of target genes [147]. STAT3 dimerization relies on the interaction between the Src-homology 2(SH2) domain of two monomers. Based on this mechanism, researchers are keen to find small-molecule inhibitors that act on the SH2 domain to block STAT3 dimerization and transcriptional activity. However, several inhibitors acting on the STAT3 SH2 domain have demonstrated limited clinical value because of the existence of structural homology between STAT family members, making obstacles for specific STAT3 inhibitors development [148, 149]. Another problem stands out that the single STAT3 protein is still transcriptionally active [150], so developing inhibitors of the STAT3 SH2 domain is not a feasible approach to fully suppress the activity of STAT3. PROTACs have a promising prospect as a novel therapeutic for the degradation of targeted protein [151]. Here we introduced a specific and potent STAT3 PROTACs. Bai et al*.* reported the first STAT3 PROTAC SD-36 (Fig. 2i) that not only could effectively and specifically degraded STAT3 and has the antiproliferative activity of leukemia and lymphoma cell lines [147]. SD-36 consists of a selective STAT3 inhibitor SI-109 and lenalidomide and is a typical successful example of how PROTACs can be applied to target challenging proteins such as transcription factors.

## Design and development of PROTACs

The degradation activity of PROTACs not only depends on the affinity of both ends to their respective target, but also relies on the formation of ternary complex that can form stable PPI. Currently, the construction of PROTACs largely relies on empirical analyses and structure–activity relationship (SAR) studies. However, synthetic difficulty presents significant limitations for rapid synthesis of a wealth of PROTAC compound libraries. By analyzing and summarizing published PROTACs structures, we will provide conventional strategies in PROTAC design to accelerate PROTACs discovery. In addition, we have listed some recently reported PROTACs that recruit traditional E3 ligases with corresponding degradation activity (Table 4).

**Table 4:** Representative compounds of PROTACs reported since 2019

Compound	Target	E3 ligase	Structure	Activity		Ref
				DC_50_	D_max_	
VZ185	BRD7/9	VHL		BRD9 DC_50_ = 1.8 nM BRD7 DC_50_ = 4.5 nM	95%	[152]
SJF-0628	BRAF^*V600E*^	VHL		SK-MEL-28 DC_50_ = 6.8 nM	≥ 95%	[153]
MS39	EGFR	VHL		HCC-827 EGFR^*e19d*^ DC_50_ = 5.0 nM H3255 EGFR^*L858R*^ DC_50_ = 3.3 nM	/	[154]
SHP2-D26	SHP2	VHL		KYSE520 DC_50_ = 6.0 nM MV4;11 DC_50_ = 2.6 nM	> 95%	[155]
PZ15227	BCL-xL	CRBN		WI38 DC_50_ = 46 nM	96%	[156]
ZB-S-29	SHP2	CRBN		MV4;11 DC_50_ = 6.0 nM	/	[157]
NR-6a	P38α/β	CRBN		T47D DC_50_ = 2.9 nM MDA-MB-231 DC_50_ = 18.4 nM	/	[158]
MS-154	EGFR	CRBN		HCC-827 EGFR^*e19d*^ DC_50_ = 11 nM H3255 EGFR^*L858R*^ DC_50_ = 25 nM	/	[154]
A1874	BRD4	MDM2		HCT116 DC_50_ = 32 nM	98%	[159]
BC5P	BTK	cIAP1		THP-1 DC_50_ = 182 nM	/	[160]
-	BCL-xL	cIAP1		/	/	[161]
MS159	NSD2	CRBN		293FT DC_50_ = 5.2 μM	> 82%	[162]

### E3 ligase and its ligand

**Fig. 3:**
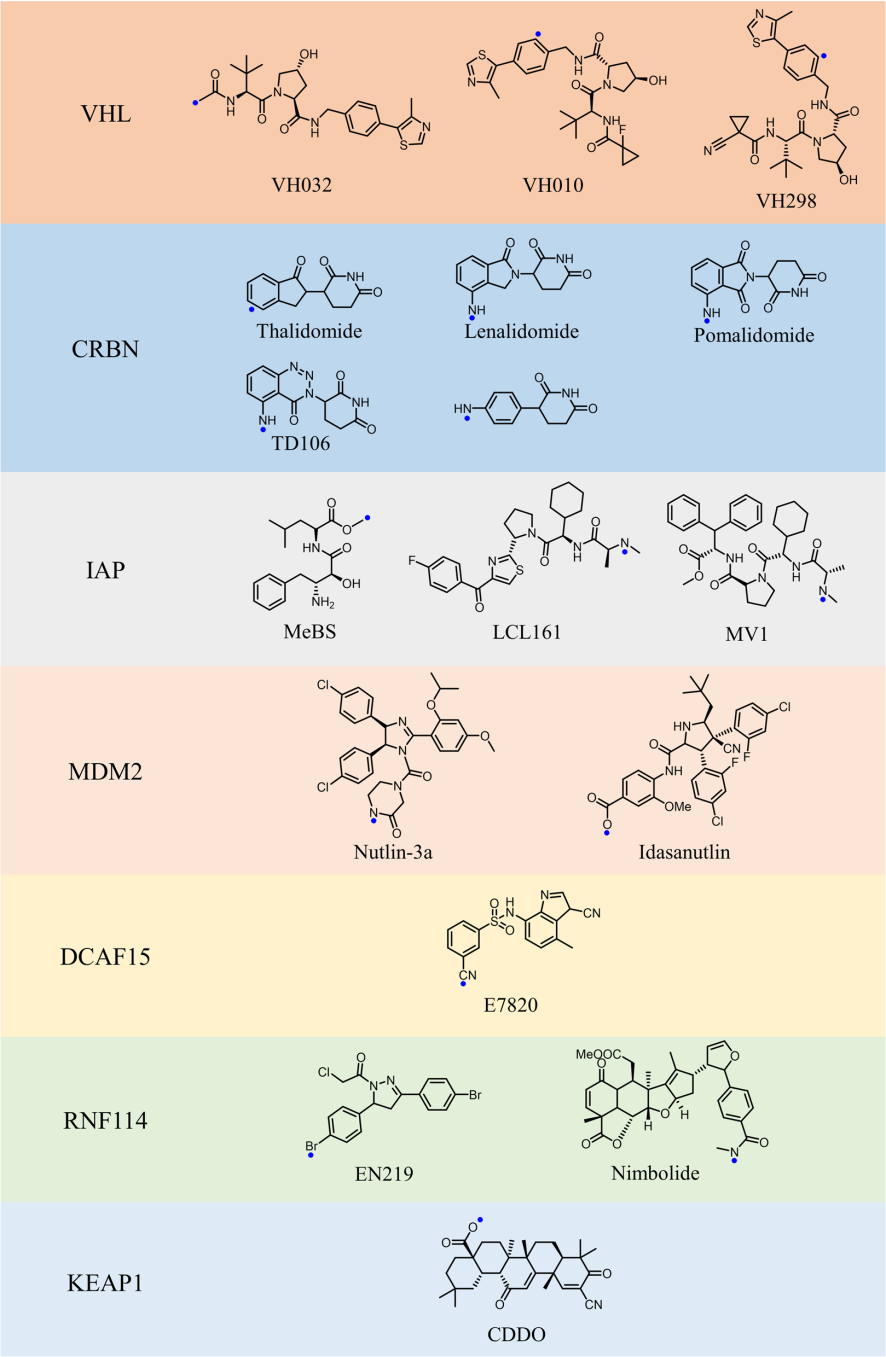
Representative small molecule ligands of E3 ligases used for PROTACs. Blue dots indicate the appropriate linker attachment site

Of the more than 600 ligases identified, only a few with small molecule ligands have been used for PROTAC targeting [163]. We list the commonly used E3 ligases and their ligands (Fig. 3). Cao et al*.* summarized and analyzed the structures of highly active PROTACs published over 20 years, and they found that CRBN, VHL, and cIAP ligands were used most frequently, of which CRBN accounted for 60.1%, VHL for 30.1%, and cIAP for 5.5% [164]. The main reason is that CRBN is widely expressed in tissues with high abundance and CRBN-based PROTACs have better degradation efficiency. In addition, CRBN ligands have better drug-like properties compared to the VHL ligand. PROTACs recruiting MDM2 and cIAP usually have high molecular weight and poor tissue permeability, indicating that the oral bioavailability may be a potential concern. Some other E3 ligases such as DCAF11 [27], DCAF15 [28], DCAF16 [29], KEAP1 [30], and RNF114 [31] etc., are less used for the following reasons: their ligands are derived from natural products with poor affinity, and are difficult to synthesize, and most of these E3 ligases are recruited by irreversible PROTACs, which have poor degradation activity and some potential toxicity. Of note, different recruited E3 ligases have been shown to induce different degrees of protein degradation [165]. The major reasons are as follow: different expression levels of E3 ligases in different cells may contribute to the different degradation efficiency. And some proteins have different degrees of selectivity for different E3 ligases. Therefore, in the process of designing the PROTACs, ligands targeting CRBN or VHL should be preferentially chosen, as these two E3 ligases have the widest range of applications. As an illustrative example, both ARV-110 and ARV-471 selected CRBN ligase as the E3 ligand. Here, we review the traditional E3 ligases and their ligands used in PROTAC design.

### Linker design strategies of PROTACs

#### Type of linkers

Maple’s group built a database containing more than 400 published PROTACs to find a general principle that has been applied in PROTAC [166]. A summary of the linker structures in the database (Table 5) reveals that the frequently used linkers in PROTACs design are PEG and (un)saturated alkane chains with varying lengths up to now [81]. Due to the facile chemical synthesis feature, alkyl linkers are often used for the synthesis of PROTAC molecules to identify the optimal linker length. However, introduction of alkyl linkers might reduce the cell permeability of PROTACs due to their high hydrophobicity. Alkyl chains containing heteroatoms (oxygen atoms or nitrogen atoms) have improved hydrophilicity over alkyl chains alone. In addition, incorporating PEG chain can enhance the solubility and uptake of PROTACs by cells. More than half of the published PROTACs structure contained alkyl and PEG motifs. Alkyl, PEG, and glycol chains are incorporated into the PROTACs to increase the flexibility. However, their introduction can affect the pharmacokinetics (PK) properties of PROTACs. In recent years, linear linkers are gradually replaced by rigid linkers, such as alkynes and saturated heterocycles (piperazine and piperidine). The incorporation of aromatic rings or alkyne chains imparts some rigidity and promotes stable ternary complex formation. It also facilitates the solubility and cell permeability of PROTAC [167]. Thus, making it orally bioavailable and clinically effective, such as ARV-110, ARV-471, and BTK PROTACs [168]. Click chemistry is commonly applied to construct PROTAC molecules in vivo, so the triazole group is chosen to link POI and E3 ligase ligand. However, it’s difficult to metabolize triazole in vivo, therefore, the introduction of triazole may help to enhance metabolic stability and prolong the durability of PROTACs [169]. The discovery process of ARV-110 is an example of great reference value in the development of PROTACs. In earlier study, AR antagonists and VH032 based ARCC-4 was discovered with efficient degradation activity. Given the lack of oral bioavailability of ARCC-4, the VHL ligand was replaced with a CRBN ligand and the linker was optimized accordingly to improve bioavailability. Then the warhead was further modified to obtain two PROTACs with superior in vivo and in vitro activity to ARCC-4, but both compounds had a high clearance rate. The activity and bioavailability were improved after switching to a rigid linker, and further optimization of the dose-escalation exposure finally led to the discovery of ARV-110 [170].

**Table 5:** Occurrence of selected linker motifs in the Maple database of published PROTACs structures [81]

Structure	Linker motif	Occurrence in Maple database structures (%)
	PEG	54
	Alkyl	31
	Other Glycol	14
	Alkyne	7
	Piperazine	4
	Piperidine	4
	Triazole	6

#### Length of linkers

The length of the linker also has a significant effect on the degradation activity of PROTAC [171]. Recently, Bemis et al*.* presented a model of linear linker length SARs studies which suggested that degraders with longer linkers are more likely to succeed in the preliminary design of PROTAC. Once the efficient PROTAC is identified, the length of the linker will be shortened step by step to identify the optimal linker length [172]. When the linker is too short, it is difficult for the 2 ligands to bind to their respective targets simultaneously because of steric hinderance effects, thus preventing the formation of ternary complex [171]. However, in case that the linker is excessively long, it will hinder the PPI, resulting in the failure of target protein ubiquitination [73]. Additionally, longer linkers have larger molecular weights, which make PROTACs less likely to cross cell membrane. Hence, the incorporation of rigid linkers, such as alkyne, piperazine and piperidine into linkers can effectively improve the pharmacokinetic profile and efficacy of PROTACs [123].

#### Choosing an appropriate linker attachment site

PROTACs have three necessary components, a warhead, an E3 ligase ligand, and a linker connecting them. Once the warhead and the E3 ligase ligands have been fixed, the selection and optimization of composition, length and attachment sites are essential factors to construct PROTACs. PROTAC molecules with suitable linkers have a significant impact on the activity and selectivity for POI degradation [54]. In general, its preferable to access the linker from the solvent-exposed position of the target protein, where it does not affect the binding affinity of its ligand. In most cases, researchers have identified the appropriate linkage position through co-crystal structure and SAR study. For warheads and E3 ligase ligands, the choice of linker attachment site requires to be considered without affecting the original affinity to its receptor. Most importantly, do not derive linkers from the critical active group of the ligand.

#### Photo-control linkers

**Fig. 4:**
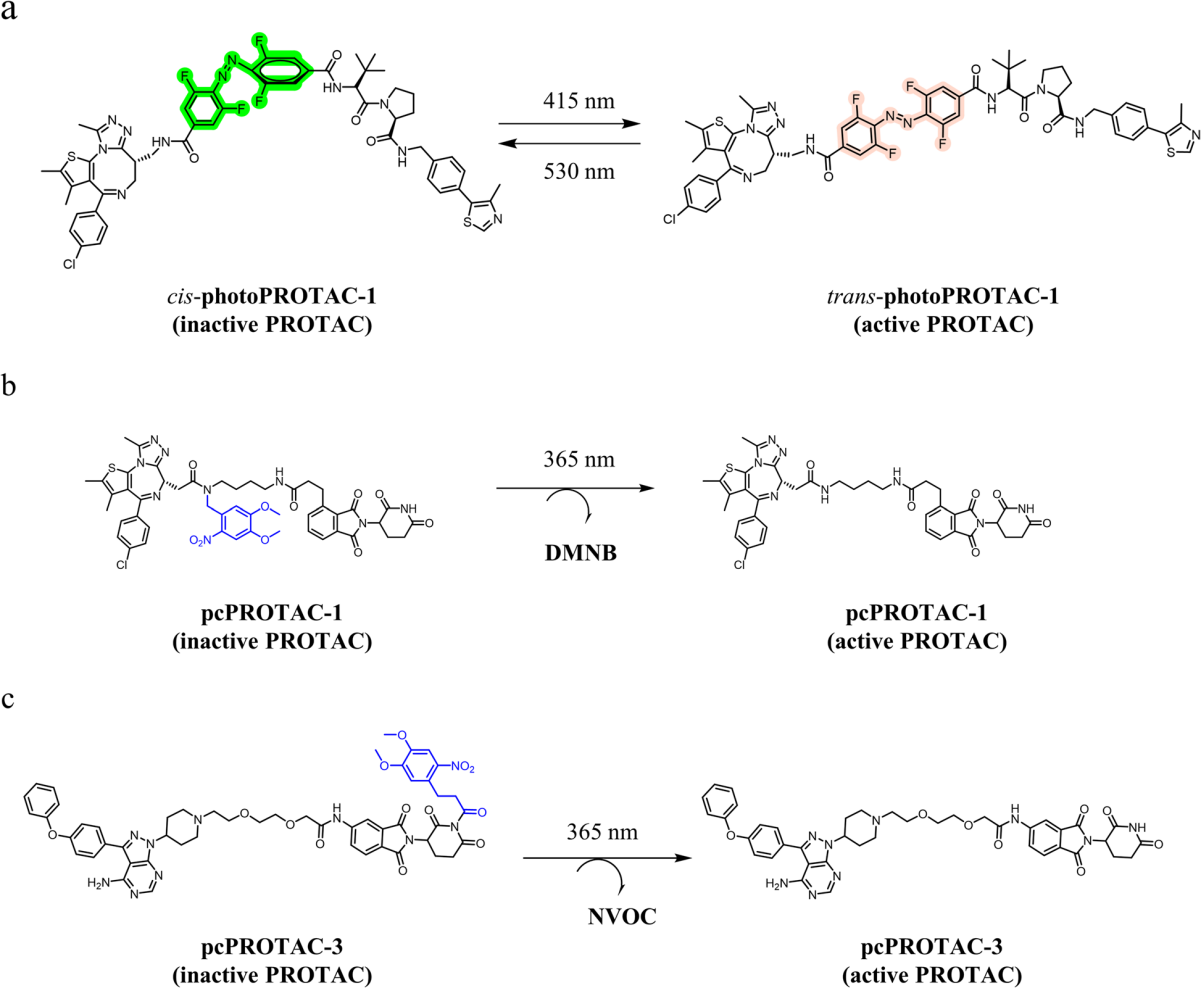
**a** photoPROTAC-1 comprising BRD-targeting JQ1 and a VHL ligand linked via a photoswitchable tetrafluoro azobenzene moiety. Light irradiation converts the inactive cis-photoPROTAC-1 into its active trans isomer, and vice versa, **b** irradiation of DMNB-protected PROTAC at 365 nm releases potent BET degrader dBET1, **c** irradiation of NVOC-protected PROTAC at 365 nm releases potent BTK degrader

Although the catalytic MOA offers fewer side effects for PROTACs over traditional small-molecule inhibitors. However, the unique catalytic feature of PROTACs also rendered inevitably adverse effects resulting from robust degradation in both normal and cancer cells. To overcome this problem, introduction of photocontrol linkers enabled the degradation of POI in a spatiotemporal manner [173, 174]. Incorporating photo-switchable azobenzene group into linker is a reversible way to control degradation activity with light. PROTAC will switch reversibly between “*cis*” and “*trans*” at different given wavelengths of irradiation, resulting in the conformation of corresponding inactive or active PROTAC. After irradiation at a certain wavelength, the inactive PROTAC will be converted to the active isoform. Only active PROTAC has the capability to form a stable ternary complex with target proteins, triggering subsequent ubiquitination and proteasome degradation. In addition, degradation also can be interrupted by switching back to the inactive form under certain wavelengths of light. Pfaff et al*.* have designed a bistable photoPROTACs using tetrafluoro azobenzene group to optically control the degradation of BET [175]. The photoPROTACs connected the VHL ligand and JQ1 together via a photoswitchable tetrafluoro azobenzene linker (Fig. 4a).Xue’s group developed a different method to optically control the degradation of target proteins through incorporation of a photocaging group to either warhead or E3 ligand to hinder the formation of a stable POI-PROTAC-E3 ligase complex [176]. Under the specified wavelength light irradiation, once the photocaging moiety is released, it can be reverted to the active conformation. In 2019, a photocaging strategy was first used in PROTAC. A photocaging moiety was conjugated to the warhead side in dBET1, creating pcPROTAC1 [176] (Fig. 4b). Under 365 nm wavelength irradiation, the 4,5-dimethoxy-2-nitrobenzyl (DMNB) was released, resulting in the formation of active dBET1. Additionally, photocaged PROTAC-3 introduced the photocaging groups into CRBN ligand side to hinder CRBN recruitment, Under 365 nm wavelength irradiation, the uncaged pcPROTAC-3 induced BTK degradation [177] (Fig. 4c). These studies proved the probability of introducing the photocaging groups to either side. However, conjugating to POI ligand would be better than E3 ligand, as it excludes the effect of the inhibitory activity that the target protein has in the absence of light exposure.

#### Clickable linkers

Click reactions are valid bioorthogonal tools for the self-assembly of PROTACs in cells, and improve the poor permeability of PROTACs. Astex Pharmaceuticals has developed smaller precursor-based intracellular CLIPTACs (In-cell click-formed proteolysis targeting chimeras, CLIPTACs) system [178]. The small molecule precursors in this system have smaller molecular weights, such as the tetrazine (Tz)-tagged thalidomide derivative (~ 572 Da) and the trans-cyclo-octene (TCO)-tagged JQ-1 derivative (~ 609 Da). Heightman’s group developed two model CLIPTACs which can be synthesized intracellularly via click reaction of trans-cyclo-octene and tetrazine precursor molecules [178]. The results showed that CLIPTACs were capable of successfully inducing the degradation of BRD4 and ERK1/2 (extracellular regulated protein kinase) in three cell lines, including HeLa, A375 and HCT116 [178]. This pioneering strategy not only improves the cell permeability and solubility of PROTACs, but also eliminates the need for linker optimization, which is more flexible and convenient as only the protein ligand fraction needs to be changed for different target protein degradation.

### Computer simulation accelerates PROTAC design

The rational design of PROTACs includes three components: warhead, E3 ligand and linker. Although the discovery process of warheads and E3 ligands is similar in nature to that of small molecules, but the design of linkers is somewhat challenging since POI and E3 ligases cannot interact in the absence of an effective PROTAC. As the importance of linker to the physicochemical properties and degradation activity of PROTACs are better understood, current research have focused on de novo PROTAC design. The length, composition, flexibility, and attachment sites of the linker all have a dramatic effect on the degradation efficiency. In addition, another design challenge arises from the fact that PROTAC molecules often have poor solubility, poor permeability, low bioavailability, and unpredictable hook effects, which hinder the clinical translation of PROTACs. Therefore, it’s urgent to discover new methods to improve the discovery efficiency of PROTACs. To accelerate the design progress of rational PROTACs, Zheng et al*.* created a novel depth-generating model (PROTAC-RL) [179]. A pair of E3 ligands and warheads are input into the model, and the designed linkers are output along with chemically feasible PROTACs having specific properties under the guidance of Reinforcement Learning (RL) [179]. Specifically, they first pre-trained a linker generation model (Proformer) based on transformer neural network. To overcome the challenge of low PROTAC training data, the model was first pre-trained through many quasi-PROTAC small molecules similar in size to PROTAC, and then the model was fine-tuned with real PROTACs set and augmented data. The Proformer was subsequently fed into a memory-based reinforcement learning framework, PROTAC-RL, and rewarded with experience to obtain PROTACs with ideal PK properties. To prove the validity, the research team identified BRD4 as POI and generated 5000 PROTACs. Relying on supercomputing capabilities, they further clustered and screened these virtual molecules. The researchers finally selected, synthesized and experimentally tested six PROTACs, three of which exhibited inhibitory activity, and one lead compound showed high anti-proliferative activity against tumor cell lines and good pharmacokinetics. Western blot assay results showed that compound 1–3 (Fig. 2j) decreased intracellular BRD4 at micromolar concentration. And all these compounds showed anti-proliferative activity against Molt4 cell line at micromolar concentrations. The entire research effort took only 49 days, indicating that the application of computer models can facilitate efficient rational PROTAC design and optimization.

## Application of PROTACs in diseases

Over the last two decades, PROTACs have demonstrated unique advantages in addressing disease associated proteins. Currently, some representative PROTACs have reached clinical trials for the treatment of cancers. Except for cancer, PROTACs also offer great advantages in the treatment of other diseases, such as neurodegenerative diseases, immune system diseases or viral infection. Here, we summarize some PROTACs targets for these diseases (Table 6).

**Table 6:** Diverse PROTACs targets on different diseases

Disease fields	Targets
Cancer	Kinase: BTK, FAK, MEK, IRAK4, BCR-ABL, EGFR, CDK, Aurora A
	Transcriptional factors: AR, ER, STAT3
	Epigenetic proteins: EZH2, BRD, HDAC, KDM5C, Sirt2, EDR5, PRMT5, NSD3, NAMPT, ENL, p300/CBP
Neurodegenerative diseases	GSK-3β, LRRK2, α-Synuclein, Tau, TRKA, TRKC, mHtt
Immune disorders	HDAC3, H-PGDS, IRAK1, IRAK3, IRAK4
Virus diseases	PEGS-2, NS3/4A, Mpro
Others	HMGCR, VEGFR2

### PROTACs targeting cancer-related targets

The indispensability of oncogenic proteins in the progression of cancer makes PROTAC particularly suitable for the treatment of cancer. Most of the current research on PROTACs focused on cancer-related targets. In the reported studies, researchers preferred kinases as degradation targets [103]. Statistically, kinases account for 45% of the total targets degraded by PROTAC [180–184], of which, more than half of PROTACs targeted RTK [185] and CMGC kinase group (CMGCs). BTK PROTACs have entered clinical trials and several compounds have shown good clinical benefits. PROTACs targeting kinases such as ALK, MEK and CDK have also been studied and investigated extensively in the literature. Besides the kinase-based PROTACs, there are still a large number of PROTACs focused on targeting nuclear receptors and epigenetic protein. So far, the most successful targets for PROTAC applications are AR and ER. Compared with kinase small-molecule inhibitors, the resistance of AR and ER is very complex and tricky. Due to the diversity of AR mutations, original inhibitor of AR, enzalutamide, may lose its inhibitory efficacy and even become a partial agonist. Therefore, PROTACs are particularly suitable for the treatment of AR related cancer, especially metastatic castrate-resistant prostate cancer.

### PROTACs for treating neurodegenerative diseases

The most common neurodegenerative disorders include Alzheimer’s disease, Huntington’s disease, and Parkinson’s disease [186]. They often occur in the elderly population and, are a class of diseases that cause cognitive impairment. Aggregation of misfolded proteins is one of the leading cause of neurodegenerative diseases, and the commonly misfolded proteins are β-amyloid, tau, alpha-synuclein, and polyglutamates [186].

Tau is an important microtubule-associated pathological protein of Alzheimer’s disease [187], which is difficult to regulate like many non-enzymatic proteins, because of the lack of active pockets. One of the most prominent features of AD and other neurodegenerative diseases is the accumulation of Tau [30]. Tau levels are higher in the brains of patients with AD than in healthy people. A high Tau level can promote its aggregation and also affect the toxicity of amyloid-β (Aβ). Thus, minimizing Tau aggregation is considered as a potential way to treat AD. Lu’s group designed and synthesized a peptide-based PROTAC bearing Keap1 E3 ligase ligand for the degradation of intracellular Tau, it showed high affinity with tau and keap1 in vitro and induced moderate degradation of Tau [30].

Huntington’s disease (HD) is caused by the variation of Huntington gene, and the abnormal mutant huntingtin (mHtt) produced by the variation that accumulates in the brain will affect neural and nerve cell function [188]. Consequently, inhibition or clearance of toxic mHtt aggregation is considered as a potential treatment modality [189]. Previous research mainly focused on the development of chemical small molecules that have inhibitory effects on mHtt aggregates. Unfortunately, because of the unclear machinery of chemical aggregation modulators, no applicable clinical results are available. Tomoshige et al. designed two small molecule PROTACs, conjugating probes for mHtt aggregates with a ligand for ubiquitin ligase cIAP1. Experimental data showed that the two compounds are capable of inducing the degradation of mHtt in living cells [190]. The effect is particularly pronounced in HD patients and mHtt with a much longer polyglutamine repeat sequence (145Q).

### PROTACs for treating immune-related diseases

#### IRAK4

Interleukin-1 receptor-associated kinase 4 (IRAK4) plays an important role in toll-like receptors (TLRs) and interleukin1 receptors (1L-1R) signaling pathways [191]. IRAK4 belongs to a family of four kinases (IRAK4, IRAK1, IRAK2, and IRAK-M) [192]. IRAK4 receives signals from the upstream TLRs as well as the 1L-1R and activates its downstream NF-κB and JNK signaling pathways, which are closely related to human inflammatory responses and cancers. After TLRs or 1L-1R receptors are activated, IRAK4 binds with MyD88 and IRAK2 through the shared death domain (DD) to form a myddosome complex. The myddosome complex performs its phosphorylation function and activates the downstream IRAK1 and related factor 6 (TRAF6), thus activating the downstream NF-κB and JNK signaling pathways to transcribe genes associated with inflammation and cellular proliferation. Overactivation or dysfunction of IRAK4 can lead to different problems accordingly. In addition to its kinase activity, IRAK4 also has scaffolding signaling. Therefore, traditional small-molecule inhibitors are unable to block all the functions of IRAK4. As a promising technology, PROTACs can eliminate all the functions of protein. In 2020, Dai et al. reported several PROTACs which could selectively degrade IRAK4 [193]. Among all of the PROTACs, only one PROTAC induced the degradation of IRAK4*.*

#### HDAC3

The histone deacetylases (HDACs) family is a class of chromatin-modifying enzymes that silence transcription via the modification of histones [194]. HDACs family consists of eighteen isoenzymes that can be divided into four types [195]. Among them, HDAC1-3 and 8 belong to class I HDACs that play a key role in cell motility, immunoregulation, and proliferation [196]. However, the structure of HDAC3 contains a well-conserved catalytic structural domain that makes selective targeting of HDAC3 challenging. In 2020, Dekker et al. reported a novel HDAC3 PROTAC HD-TAC7 (Fig. 2k), which consists of CRBN ligand pomalidomide and selective class I HDAC inhibitors o-aminoanilide [197]. HD-TAC7 has a medium degradation potency but no effect on HDAC1 and HDAC 2. This year, Liao et al*.* unraveled VHL-based PROTAC XZ9002 (Fig. 2k) that could specifically degrade HDAC3 and inhibit tumor cell activity [198].

### PROTACs targeting virus-related targets

It has been thought that PROTACs also can be applied in the antivirus field to reduce susceptibility to resistance mutations. With the drug resistance of conventional antiviral drugs, the effect of clinical treatment began to gradually deteriorate. Recent study leveraged PROTACs to develop a chemical knock-down antiviral to induce degradation of viral proteins. Wispelaere et al*.* designed a PROTAC which consists of a reversible-covalent inhibitor telaprevir that binds to the hepatitis C virus (HCV) protease active site and a ligand for CRBN ligase [199]. The compound DGY-08-097 (Fig. 2l), not only inhibits but also degrades the HCV NS3/4A protease, exhibiting efficiency in a cellular infection model [199].

Severe acute respiratory syndrome coronavirus 2 (SARS-CoV-2) is a serious threat to the lives and health of people around the world since its outbreak in 2019 [200]. Despite the fact that several vaccines have been designed worldwide against COVID-19, the high mutagenicity of the virus limits the effectiveness of vaccine. In 2021, Desantis et al. designed a series of indomethacin-based PROTACs pan-coronavirus antiviral agents [201]. Indomethacin (INM) has antiviral activity, but the mechanism behind it is not known. The antiviral activity of INM against SARS-CoV-2 probably came from its inhibitory activity to human prostaglandin E synthase type 2 (PGES-2). Previous study has reported that INM has inhibitory activity of PGES-2 in the nanomolar concentration [202, 203]. The PGES-2 has been shown to have an interaction with the NSP7 protein of SARS-CoV-2 [204, 205]. And the interaction of NSP7 with PGES-2 was also present in other coronaviruses [204, 206], suggesting that targeting PGES-2 may be a potential approach for INM-based antiviral PROTACs design. Desantis et al. designed four INM-based PROTACs, but the biological evaluation results showed that only two compounds were about 4.5-fold more potent than INM, as well as a wide-spectrum antiviral activity against the β-coronavirus HCoV-OC43 and α-coronavirus HCoV-229E [201].

### Other PROTACs

In 2020, Rao et al. reported the first PROTAC of HMG-CoA reductase (HMGCR), which is the rate-limiting enzyme in the cholesterol biosynthetic pathway [207, 208]. They synthesized a series of PROTACs by tethering Atorvastatin and CRBN ligands. After optimization and screening, they ultimately found the most potent degrader P22A (Fig. 2m) with DC_50_ of 0.1 μM [209]. This PROTAC stressed the potential application for the treatment of hypercholesterolemia and cardiovascular disease. In addition, PROTACs are a promising therapeutic approach in other non-oncoproteins. Li et al. reported the first PROTAC that induced degradation of α_1A_-adrenergic receptor (α_1A_-AR) and is also the first PROTAC for G protein-coupled receptors (GPCRs) [210]. They connected α_1A_-AR inhibitor prazosin with pomalidomide by different linkers and finally found the potent compound 9c (Fig. 2m). 9c could inhibit the proliferation of PC-3 cells and cause tumor growth slowdown, which provided a new strategy for the treatment of prostate cancer. Hu et al. presented the first PROTAC of indoleamine 2,3-dioxygenase 1 (IDO1) [65]. IDO1 has been extensively reported as key immune checkpoint, which overexpressed in multiple cancers [211]. Hu et al. discovered the first PROTAC 2c (Fig. 2m) which induced the pronounced and sustained degradation of IDO1. Si et al. showed that PROTAC of hematopoietic progenitor kinase1 (HPK1) helped to improve CAR-T cell-based immunotherapy [212]. PROTAC technology is so widespread in the field of disease treatment, making it a powerful tool for drug discovery.

## Disadvantages and future challenges of PROTAC

As an emerging technology, PROTAC has attracted great attention from academia and the pharmaceutical industry. The development of any new technology comes with various opportunities and challenges, and PROTAC is no exception. The prospect of potential opportunities and challenges for PROTAC will contribute to the research and development of targeted protein-degrading drugs. Although PROTAC has unique advantages over other drug discovery paradigm, it also has some disadvantages, which bring nonnegligible issues and challenges:

- Pharmaceutical property: PROTAC molecule is more complex than traditional small-molecule drugs and has more potential metabolic sites, which affects the metabolic stability of PROTAC molecules. At the same time, traditional small-molecule inhibitors generally follow the “Rule of Five”, but most of the reported PROTACs tend to have a molecular weight greater than 700, resulting in poor permeability, low solubility and unsatisfactory oral bioavailability [213]. Therefore, how to improve physicochemical properties of PROTAC molecule will be the key to its successful drug formation if “the Rule of Five” are not satisfied.
- Resistance: First, PROTACs can cause drug resistance through the change in the genome of the core component of the E3 ligase complex. Significantly reduced expression of CRBN gene or CUL2 gene can also cause resistance to PROTACs [214, 215]. Studies have shown that deletion of the CRBN genome is the main reason for myeloma cells to develop resistance to IMiDs. Secondly, the action of PROTAC depends on specific E3 ligase subtype, and the expression of specific E3 ligase limits the application of PROTAC in different cell types. Although the human genome encodes hundreds of E3 ubiquitin ligases, only a few E3 ligases and small molecule ligands have been used for PROTACs. Therefore, finding more kinds of E3 ligases for the research and development of PROTAC drugs might be the way to solve drug resistance [216].
- “Hook effect” and “Off target”: How to avoid Hook effect and off-target effect is also a major challenge for PROTAC drugs development. The higher the concentration of drugs, the better degradation effect is not necessarily for PROTACs, which is often referred to as the “Hook effect”. In the research of PROTACs, it has been found that significantly higher concentration than DC_50_ will result in self-inhibition effect to compensate degradation efficiency, called “Hook effect” [217, 218]. In addition, the mechanism of off-target effects of PROTACs molecules have not been fully understood [219]. PROTACs can completely degrade target protein, thus inhibit all functions of target protein. However, in this process, normal protein may be accidentally injured, off-target effect and toxicity are also one of the biggest challenges. For example, studies have shown that thalidomide derivatives can cause degradation of transcription factors such as IKZF1, IKZF3 and GSTP1 [214]. Further studies found that the degradation of thalidomide derivatives on transcription factors such as GSPT1 was due to their “molecular glue” effect.
- Target selection: To date, what targets are appropriate for PROTAC technology to achieve better benefits than small-molecule inhibitors are not fully understood and most of the target proteins of the PROTACs are part of the “druggable” protein. In fact, one of the greatest advantages of PROTAC technology is its potential to handle “undruggable” target. Because PROTAC technology only needs temporarily mediate the formation of ternary complexes, low affinity POI ligands can be incorporated into PROTAC molecules. Unfortunately, there are only few PROTAC molecules targeting “undruggable” proteins to date. Therefore, another challenge for PROTACs is the need to develop more molecules that target “undruggable” proteins and thus embody the advantages of PROTAC technology.

## Discussion and conclusion

As an emerging paradigm for drug discovery, PROTACs have attracted great attention from academia and industry. Although PROTAC technology has many advantages in drug development, there are still many obstacles and challenges in the process of discovery and clinical application, such as off-target, cell permeability, stability, and large molecular weight, etc. In addition, the issues of oral bioavailability and drug integrity are also ongoing challenges for PROTAC drug development. It is worth noting that PROTAC still has many advantages in clinical application compared with other traditional small-molecule inhibitors. First, PROTAC plays a role by inducing the degradation of pathogenic proteins, so it can promote the degradation of multiple rounds of target proteins, assisting to eliminate off-target effects and accumulation of drug targets. PROTAC can also degrade some proteins that are considered “undruggable”, such as transcription factors. Secondly, PROTAC has the advantages of improving selectivity and specificity, overcoming drug resistance. In short, the current status of PROTAC drug development is the coexistence of both advantages and disadvantages, but how to solve these problems will be the key to the success of PROTAC drug development.

The discovery of efficient PROTAC molecules is a time-consuming and challenging process, such as the optimization of linker length and structure. It is urgent to summarize a general method for designing efficient PROTAC molecules. At present, the design and optimization of PROTAC mainly focus on the structure–activity relationships research of POI ligands and linker. Among them, linker is not only critical to the degradation activity of PROTACs, but also greatly affects the membrane permeability, metabolic stability and drug availability. Therefore, how to effectively design and link POI and E3 ligands is the key to the molecular design of PROTACs. Up to now, the principles guiding the design of linker, including length and composition, have not been fully understood. On the other hand, photo-PROTAC designed based on “photo control linkers” also has some advantages over traditional drugs, which is also introduced in this article. It is expected that the newly emerging photo-PRTOAC can become a leading way among PROTAC drugs. In this review, we summarized the general principles in the design of PROTAC, providing a systematic understanding for the research and design of PROTACs. In addition, E3 ligase is also crucial in the composition of the ternary complex. However, among the hundreds of E3 ligases encoded by the human genome, only a few E3 ligases are used in PROTACs, and the progress in discovering new E3 ligases and their ligands is far behind the research of PROTACs. So far, the majority of PROTACs induce target protein degradation by recruiting E3 ligases CRBN, VHL, MDM2 and IAP, and the research on PROTACs by only these E3 ligases is still far from enough. Therefore, it is necessary to explore more novel E3 ligases to accelerate the development of PROTACs. However, it can be predicted that the number of E3 ligands may increase significantly in the future, which will provide more options for the design of PROTACs.

PROTAC technology has been developed for nearly 20 years, and some molecules have entered clinical trials, which reveals the huge therapeutic potential of PROTACs in tumor, immune disease, neurodegenerative disease, cardiovascular disease and viral infection. There are also studies around the world using this technology to treat COVID-19. So far, two PROTAC drugs ARV-110 and ARV-471 have entered the phase II clinical trial, which are used to treat prostate cancer and breast cancer respectively. Although more than ten drugs are in clinical trials, clinical research data are still insufficient, and more clinical studies are needed to prove the prospects of PROTAC technology. With the deepening of research, these obstacles will be basically solved in the near future. Once more drugs enter the clinical application, it will open a new era of drug research and development.

Although there are still many obstacles and challenges to be overcame, PROTACs have great therapeutic potential with its unique advantages. It is believed that in the future, with the development of technology and in-depth research, the design and synthesis of PROTACs will be gradually optimized, which will eventually open up a broad road for the treatment of various diseases, and is expected to provide clinical therapeutic benefits in the near future. In a word, PROTAC technology not only provides a powerful tool for the research in the field of pharmaceutical chemistry, but also brings great hope for the development of clinical drugs in the future.

## Data Availability

Not applicable.

## Abbreviations

PROTACs: Proteolysis targeting chimeras
POI: Protein of interest
UPS: Ubiquitin-protease system
MetAP-2: Methionine aminopeptidase-2
β-TRCP: β-Transducin repeat-containing protein
ER: Estrogen
AR: Androgen
DHT: Dihydrotestosterone
VHL: Von Hippel-Lindau
FKBP12: FK506 binding protein 12
MDM2: Mouse double minute 2
cIAP: Cell inhibitor of apoptosis protein
CRBN: Cereblon
IMiDs: Immunomodulatory drugs
BL: Burkitt’s lymphoma
Hyp: Hydroxyproline
ALK: Anaplastic lymphoma kinase
FAK: Focal adhesion kinase
RNAi: RNA interference
SGK: Serum and glucocorticoid-induced protein kinase
PI3K: Phosphoinositide 3-kinase
MOA: Mode of action
RTK: Receptor tyrosine kinase
HIF1α: Hypoxia-inducible factor-1α
PEG: Polyethylene glycol
BTK: Bruton’s tyrosine kinase
BCR: B cell receptor
C481: Cysteine481
WT: Wild type
IKZF3: Ikaros family zinc finger 3
PBMCs: Peripheral blood mononuclear cells
CLL: Chronic lymphocytic leukemia
NRs: Nuclear receptors
NLS: Nuclear localization signal
MTD: Maximum tolerated dose
RP2D: Recommended phase 2 dose
mCRPC: Metastatic castrate-resistant prostate cancer
PSA: Prostate specific antigen
ER+: Estrogen receptor-positive
HER2+: Human epidermal growth factor receptor 2 positive
SERD: Selective estrogen receptor degraders
TFs: Transcriptional factors
STAT3: Signal transducer and activator of transcription 3
SH2: Src-homology 2
PPI: Protein–protein interaction
SAR: Structure–activity relationship
DMNB: 4,5-Dimethoxy-2-nitrobenzyl
CLIPTACs: In-cell click-formed proteolysis targeting chimeras
Tz: Tetrazine
TCO: Trans-cyclo-octene
ERK1/2: Extracellular regulated protein kinase
CMGCs: CMGC kinase group
AD: Alzheimer’s disease
Aβ: Amyloid-β
HD: Huntington’s disease
mHtt: Mutant huntingtin
IRAK4: Interleukin-1 receptor-associated kinase 4
TLRs: Toll-like receptors
1L-1R: Interleukin1 receptors
DD: Death domain
HDACs: Histone deacetylases
HCV: Hepatitis C virus
SARS-CoV-2: Severe acute respiratory syndrome coronavirus 2
INM: Indomethacin
PGES-2: Human prostaglandin E synthase type 2
HMGCR: HMG-CoA reductase
RL: Reinforcement Learning
MWs: Molecular weights
BRD4: Bromodomain-containing protein 4
α_1A_-AR: α_1A_-adrenergic receptor
IDO1: Indoleamine 2,3-dioxygenase 1
HPK1: Hematopoietic progenitor kinase1
GPCRs: G protein-coupled receptors
EGFR: Epidermal growth factor receptor
TPD: Targeted protein degradation
RAS: RAt Sarcoma
LYTACs: Lysosome-targeting chimeras
AUTACs: Autophagy-targeting chimeras
AbTACs: Antibody-based PROTACs
LTRs: Lysosome-targeting receptors
M6Pn: Poly-serine-O-mannose-6-phosphonate
GalNAC: N-acetyl galactosamine
PD-L1: Programmed death protein ligand 1
